# Lactate and Risk of Incident Diabetes in a Case-Cohort of the Atherosclerosis Risk in Communities (ARIC) Study

**DOI:** 10.1371/journal.pone.0055113

**Published:** 2013-01-30

**Authors:** Stephen P. Juraschek, Ghanshyam Palamaner Subash Shantha, Audrey Y. Chu, Edgar R. Miller, Eliseo Guallar, Ron C. Hoogeveen, Christie M. Ballantyne, Frederick L. Brancati, Maria Inês Schmidt, James S. Pankow, J. Hunter Young

**Affiliations:** 1 The Johns Hopkins University, School of Medicine, Baltimore, Maryland, United States of America; 2 The Johns Hopkins Bloomberg School of Public Health, and The Welch Center for Prevention, Epidemiology and Clinical Research, Johns Hopkins Medical Institutions, Baltimore, Maryland, United States of America; 3 Division of Preventive Medicine, Brigham and Women’s Hospital, Boston, Massachusetts, United States of America; 4 Department of Medicine, Johns Hopkins Medical Institutions, Baltimore, Maryland, United States of America; 5 Section of Atherosclerosis and Vascular Medicine, Department of Medicine, Baylor College of Medicine, Houston, Texas, United States of America; 6 Center for Cardiovascular Disease Prevention, Methodist DeBakey Heart Center, Houston, Texas, United States of America; 7 Graduate Studies Program in Epidemiology, School of Medicine, Federal University of Rio Grande do Sul, Porto Alegre, Brazil; 8 Department of Epidemiology, Gillings School of Global Public Health, University of North Carolina at Chapel Hill, Chapel Hill, North Carolina, United States of America; 9 Division of Epidemiology and Community Health, School of Public Health, University of Minnesota, Minneapolis, Minnesota, United States of America; German Diabetes Center, Leibniz Center for Diabetes Research at Heinrich Heine University Duesseldorf, Germany

## Abstract

**Background:**

Oxidative capacity is decreased in type 2 diabetes. Whether decreased oxidative capacity is a cause or consequence of diabetes is unknown. Our purpose is to evaluate whether lactate, a marker of oxidative capacity, is associated with incident diabetes.

**Methods and Findings:**

We conducted a case-cohort study in the Atherosclerosis Risk in Communities (ARIC) study at year 9 of follow-up. We evaluated lactate’s association with diabetes risk factors at baseline and estimated the hazard ratio for incident diabetes by quartiles of plasma lactate in 544 incident diabetic cases and 533 non-cases. Plasma lactate showed a graded positive relationship with fasting glucose and insulin (*P*<0.001). The relative hazard for incident diabetes increased across lactate quartiles (*P*-trend ≤0.001). Following adjustment for demographic factors, medical history, physical activity, adiposity, and serum lipids, the hazard ratio in the highest quartile was 2.05 times the hazard in the lowest quartile (95% CI: 1.28, 3.28). After including fasting glucose and insulin the association became non-significant.

**Conclusions:**

Lactate, an indicator of oxidative capacity, predicts incident diabetes independent of many other risk factors and is strongly related to markers of insulin resistance. Future studies should evaluate the temporal relationship between elevated lactate and impaired fasting glucose and insulin resistance.

## Introduction

In the United States, the prevalence of type 2 diabetes is high [1] and its incidence is increasing due to rising levels of adiposity. Prior to the clinical diagnosis of diabetes, individuals undergo a period of subclinical insulin resistance [2]. Accumulating evidence suggests that low oxidative capacity due to impaired mitochondrial oxidative phosphorylation [3] is associated with insulin resistance and type 2 diabetes. It is unclear, however, whether low oxidative capacity is a cause or consequence of diabetes [4]. This question can be addressed using markers of oxidative capacity in longitudinal studies of incident diabetes.

Blood lactate is a measure of the gap between energy expenditure and oxidative capacity. Lactate is produced by anaerobic glycolysis in muscle, adipose, and other tissues. Anaerobic glycolysis, and, therefore, lactate production increase when energy demand exceeds mitochondrial oxidative capacity. Traditionally, blood lactate is used as a clinical marker of ischemia and as a measure of fitness among exercising individuals. Lactate variation in resting individuals may also be informative, however. Elevated lactate may be observed with mitochondrial impairments in oxidative phosphorylation [5], [6] and several clinical studies have demonstrated that lactate is closely related to insulin resistance [7]–[9]. In addition, a number of observational studies have shown that lactate is associated fasting glucose, fasting insulin, and the prevalence of type 2 diabetes [10]–[12]. These studies, however, were limited by cross-sectional designs. Only one prospective study in white men studied the association between plasma lactate levels and incident diabetes. This study showed that the highest quintile of lactate at baseline was associated with 2.4 (95% CI: 1.0–5.9) times the risk of developing diabetes in the lowest quintile [13]. Therefore, we examined the longitudinal association of plasma lactate with subsequent development of type 2 diabetes in a biracial cohort of middle-aged men and women using a case-cohort design.

## Materials and Methods

### Study Design

ARIC is a community-based prospective cohort study of 15,792 adults, ages 45–64. ARIC participants were identified by probability sampling from 4 U.S. communities (Forsyth County, North Carolina; Jackson, Mississippi; suburban Minneapolis, Minnesota; and Washington County, Maryland) and were enrolled between 1987 and 1989 [14]–[16]. Study participants returned for three follow-up visits in 1990–1992, 1993–1995, and 1996–1998, during which time incident diabetes was determined. Response rates for each ARIC visit were 60% (visit 1), 93% (visit 2), 86% (visit 3), and 80% (visit 4). The study protocol was approved by institutional review boards at each clinical site, namely, the IRBs of the University of Minnesota, Johns Hopkins University, University of North Carolina, University of Mississippi Medical Center, and Wake Forest University. Written consent was obtained from all ARIC participants.

We used a case-cohort design described previously [17]. We excluded participants with a diagnosis of diabetes at baseline (n = 2,018), members of ethnic groups with a small sample size (n = 95), participants whose diabetes status could not be determined in any of the three follow-up visits (n = 879), participants lacking a valid diabetes determination (n = 26), participants with missing plasma samples, anthropometic data, or related covariate data (hypertension, smoking, etc.) at baseline (n = 2,526), and non-fasting individuals at baseline (n = 212). Of the remaining 9,740 subjects, 1,105 developed diabetes during the 9-yr follow-up period. From this population, we selected a random cohort sample (n = 637) and a random incident diabetes case sample (n = 552) according to strata determined by race. Among those in the random cohort sample, 94 (14.8%) developed diabetes during the follow-up period. Of this population, an additional 7 non-case participants in the random cohort sample and 11 participants in the diabetes case sample did not have a lactate measurement and were excluded. After these exclusions, baseline covariates were compared between both the case-cohort and the complete cohort of ARIC visit 1 and found to be nearly identical.

### Plasma Lactate

We measured plasma lactate in stored samples originally collected in 1987–1989 and frozen at −70°C. Lactate was quantified with a Roche Hitachi 911 auto-analyzer, using an enzymatic reaction that converts lactate to pyruvate [18]. Quality control assessments of the assays suggested an inter-assay coefficient of reliability of 0.93 and a coefficient of variation of 9.2% [12].

### Primary Outcome: Incident Diabetes

Participants were assessed for a diagnosis of diabetes at baseline and at each of the three follow-up visits. Incident diabetes was classified as a diabetic case if a participant fulfilled any one of the following conditions: (1) a fasting glucose ≥7.0 mmol/liter, (2) a nonfasting glucose ≥11.1 mmol/liter, (3) use of a diabetes medication, or (4) self-reported physician diagnosis. For individuals diagnosed by a glucose measurement, the date of diagnosis was estimated under the assumption that glucose increased at a linear rate between visits. This linear approximation was used to estimate the time of actual diabetes onset. Similarly, the date of diagnosis for cases identified by medication use or physician diagnosis was estimated assuming a linear increase in glucose from their last diabetes-free visit [19]. As a sensitivity analysis, we restricted our diagnosis of incident diabetes to diabetes medication use or self-report of physician diagnosis, excluding the subclinical cases of diabetes indentified by glucose measures.

### Other Covariates

We included a number of covariates assessed at the baseline visit of ARIC that were known to be associated with diabetes. Definitions and methods for the assessment of these covariates were described previously [20], [21]. In brief, the following laboratory measurements were treated as continuous variables: low density lipoprotein (LDL) cholesterol, high density lipoprotein (HDL) cholesterol, triglycerides, fasting glucose, and fasting insulin. Triglycerides and insulin were log-base 10 transformed to normalize their distributions. We also determined the homeostatic model assessment insulin resistance index (HOMA-IR) by ((fasting insulin in pmol/L)/6.945*(fasting glucose in mg/dL))/405.

With regard to physical examination covariates, we determined body mass index (kg/m^2^) from height and weight measurements and measured waist circumference at the umbilical level. Smoking status (never, current, former), education (<12 years, ≥12 years), and leisure time physical activity index (ordinal integer ranging from 1 to 4) were based on replies to the Baecke Physical Activity questionnaire. Prevalent coronary heart disease (yes or no) was based on past history of myocardial infarction, heart or arterial surgery, coronary bypass, or angioplasty. Parental history of diabetes (yes or no) was defined by a diagnosis of diabetes in a participant’s father or mother. Hypertension (yes or no) was defined as either a systolic blood pressure measurement ≥140 mmHg, a diastolic blood pressure measurement ≥90 mmHg, or use of high blood pressure medications in the past 2 weeks.

### Statistical Analysis

All analyses and plots were weighted to account for the random cohort sample and the random case sample using the svy command and pweights in Stata 11.1 (StataCorp LP, College Station, TX), which entail inverse weighting of the observations according to the case-cohort sampling design [20]. Proportions and mean baseline characteristics of the study participants were reported by lactate quartiles, where lactate quartile categories were based on measurements in the random cohort sample. Standard errors were estimated using the Taylor series (linearization) method. We performed a cross-sectional analysis of the association between log-base 10 transformed lactate and physiologic correlates of insulin resistance, namely, BMI, waist circumference, log-base 10 transformed triglycerides, HDL cholesterol, fasting glucose, and log-base 10 transformed fasting insulin. Comparisons were made using linear regression and Pearson’s correlation coefficients. The HOMA-IR was plotted by lactate quartiles to further examine the relationship between lactate and lipids or glycemia. The *P*-value for the trend between HOMA-IR and lactate quartiles were calculated with linear regression, treating the median value of each lactate quartile as an ordinal variable.

A Kaplan-Meier cumulative incidence plot was utilized to visualize the relationship between baseline lactate measurements and risk of incident diabetes, using follow-up time as the time axis. A Wald test was used to evaluate for a trend across quartiles of lactate. The association between lactate quartiles and incident diabetes was further examined in nested Cox proportional hazards models that (1) addressed factors associated with both diabetes and lactate, and (2) explored the metabolic pathway relating lactate with incident diabetes by adjusting for the same factors above along with insulin and glucose. In the first series of models, we adjusted for age, gender, race, ARIC center, and education (Model 1); a diagnosis of hypertension, prevalent coronary heart disease, smoking status, leisure index, and parental history of diabetes (Model 2); BMI and waist circumference (Model 3); and log10-transformed triglycerides, LDL cholesterol, and HDL cholesterol (Model 4). In our metabolic pathway models, we adjusted for fasting glucose (Model 5a), fasting insulin (Model 5b) and both (Model 5c) in addition to the preceding covariates. Furthermore, we generated histograms of the distribution of lactate among diabetic cases and noncases and overlaid a restricted cubic spline based on Model 4 with knots at each lactate quartile to visualize the risk association.

## Results

The geometric mean for plasma lactate in the entire study sample (N = 1,077) was 7.67 mg/dL (IQR: 5.80 to 9.70 mg/dL; max: 36.1 mg/dL) (**Table 1**); plasma lactate was less than 18 mg/dL (∼2 mmol/L) in 97% of the population. Age was not related to lactate concentration (*P* = 0.55). However, the proportion of African Americans and men increased across lactate quartiles (*P*-trends of 0.03 and <0.001, respectively). The prevalence of hypertension was also greater in higher lactate quartiles, ranging from 13.9% to 34.2% (*P*-trend <0.001). Lifestyle characteristics, i.e. leisure physical activity index, smoking status, or education attainment, prevalent coronary heart disease, and parental history of diabetes were not associated with lactate quartiles.

**Table 1:** Baseline (visit 1) characteristics of ARIC participants according to quartiles of plasma lactate.

			Plasma Lactate Level (mg/dL)*				
			≤5.8 (N = 190†)	5.8–7.3 (N = 226†)	7.3–9.7 (N = 329†)	>9.7 (N = 332†)	
		Overall (N = 1,077†)	Mean or % (SE or IQR)	Mean or % (SE or IQR)	Mean or % (SE or IQR)	Mean or % (SE or IQR)	*P* for Trend§
Plasma lactate, mg/dL‡		7.7 (5.8 to 9.7)	5.0 (4.6 to 5.5)	6.4 (6.1 to 6.9)	8.3 (7.7 to 8.8)	13.1 (10.9 to 14.7)	<0.001
Age, y		52. 8 (0.2)	52.9 (0.5)	53.0 (0.5)	52.7 (0.5)	52.6 (0.5)	0.55
Black, %		19.9 (0.7)	11.8 (0.9)	19.6 (1.4)	24.0 (1.6)	23.9 (1.8)	0.03
Men, %		36.0 (2.1)	25.8 (4.0)	30.6 (4.2)	38.2 (3.9)	49.4 (4.2)	<0.001
Education beyond high school, %		43.7 (2.1)	38.2 (4.4)	49.2 (4.5)	43.5 (4.0)	44.3 (4.2)	0.67
Hypertension, %		26.3 (1.8)	13.9 (2.8)	28.5 (3.9)	28.8 (3.3)	34.2 (3.8)	<0.001
Prevalent coronary heart disease, %		2.5 (0.7)	0.0∧	2.6 (1.5)	4.1 (1.6)	3.1 (1.5)	0.10
Smoking Status							
Never, %		48.2 (2.2)	52.0 (4.6)	42.0 (4.5)	50.1 (4.0)	48.3 (4.2)	0.98
Former, %		31.4 (2.0)	27.9 (4.1)	30.56 (4.2)	32.5 (3.8)	34.4 (4.0)	0.26
Current, %		20.4 (1.7)	20.1 (3.7)	27.4 (4.0)	17.5 (2.9)	17.3 (3.1)	0.19
Leisure index		2.4 (0.02)	2.4 (0.04)	2.3 (0.05)	2.3 (0.04)	2.4 (0.04)	0.99
Parental history of diabetes, %		24.4 (1.9)	25.2 (4.0)	20.7 (3.7)	25.4 (3.6)	25.9 (3.9)	0.65
Body mass index, kg/m^2^		27.2 (0.2)	26.1 (0.4)	26.3 (0.4)	28.2 (0.4)	28.2 (0.4)	<0.001
Waist circumference, cm		94.8 (0.5)	92.2 (1.0)	92.4 (1.0)	96.9 (1.0)	97.4 (1.1)	<0.001
Triglycerides, mmol/L**		1.2 (0.8 to 1.7)	1.0 (0.8 to 1.3)	1.1 (0.8 to 1.4)	1.3 (0.9 to 1.7)	1.4 (0.9 to 2.0)	<0.001
LDL cholesterol level, mmol/L		3.4 (0.04)	3.4 (0.1)	3.4 (0.1)	3.6 (0.1)	3.4 (0.1)	0.38
HDL cholesterol level, mmol/L		1.4 (0.02)	1.5 (0.04)	1.4 (0.04)	1.3 (0.03)	1.4 (0.04)	0.04
Fasting glucose, mg/dL		98.2 (0.4)	94.8 (0.7)	96.8 (0.8)	99.2 (0.6)	101.8 (0.7)	<0.001
Fasting insulin, pmol/L**		57.2 (35.9 to 86.1)	42.7 (28.7 to 64.6)	47.3 (28.7 to 71.8)	70.4 (50.2 to 100.5)	73.5 (50.2 to 114.8)	<0.001

* The ranges of the plasma lactate quartiles were determined using specimens from the weighted random cohort sample.

† Represents the maximum number of participants in each category. Actual number may vary due to missing data.

‡ Plasma lactate mg/dL may be converted to mmol/L by multiplying by 0.111.

§ P-trend evaluated with linear or logistic regression using the median lactate value for each quartile as an ordinal variable.

∧ There were no participants with coronary heart disease in quartile 1. SE not calculated due to small sample size.

** Represents geometric mean and interquartile range.

Note: LDL represents low density lipoprotein. HDL represents high density lipoprotein.

**Figure 1:**
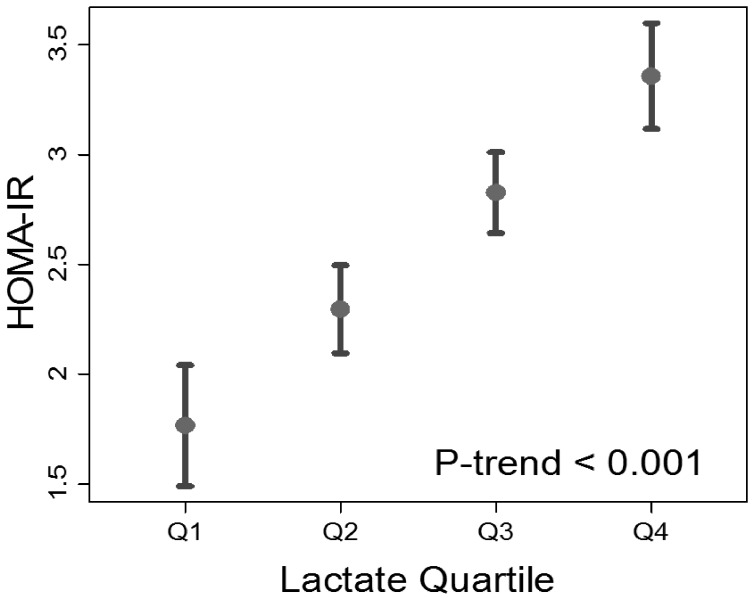
Mean homeostatic model assessment (HOMA-IR) with 95% confidence intervals by baseline plasma lactate quartile.

Higher lactate was associated with both BMI and waist circumference (both *P*<0.001) (**Table 1**). With regard to lipids, LDL cholesterol did not vary significantly across lactate quartiles (*P* = 0.38), while triglycerides concentrations were greater, and HDL levels lower, with successive lactate quartiles (*P* for trend of <0.001 and 0.04, respectively) (**Table 1**). Fasting glucose and fasting insulin also demonstrated significant positive trends by lactate quartile (both *P*<0.001) (**Table 1**). In linear regression, BMI, waist circumference, triglycerides, fasting glucose and fasting insulin were also positively associated with plasma lactate, while HDL cholesterol was negatively associated with plasma lactate (**Table 2**). Similarly, the HOMA-IR (**Figure 1**) was higher with successive lactate quartiles (*P*<0.001).

**Table 2:** Percent change in plasma lactate per 1 unit increase in physiologic correlates of insulin resistance and Pearson correlation coefficient between baseline characteristics and log-base 10 transformed lactate.

	Percent Change Per 1 Unit Increase*	*P*	Pearson’s Coefficient
Body mass index, kg/m^2^	1.2%	<0.001	0.15
Waist circumference, cm	0.3%	<0.001	0.14
Log_10_(Triglycerides, mmol/L)	53.0%	<0.001	0.22
HDL cholesterol, mmol/L	−5.9%	0.04	−0.11
Fasting glucose, mg/dL	1.0%	<0.001	0.26
Log_10_(Fasting insulin, pmol/L)	20.3%	<0.001	0.31

* Adjusted for age, gender, race, and center.

Note: HDL represents high density lipoprotein.

**Figure 2:**
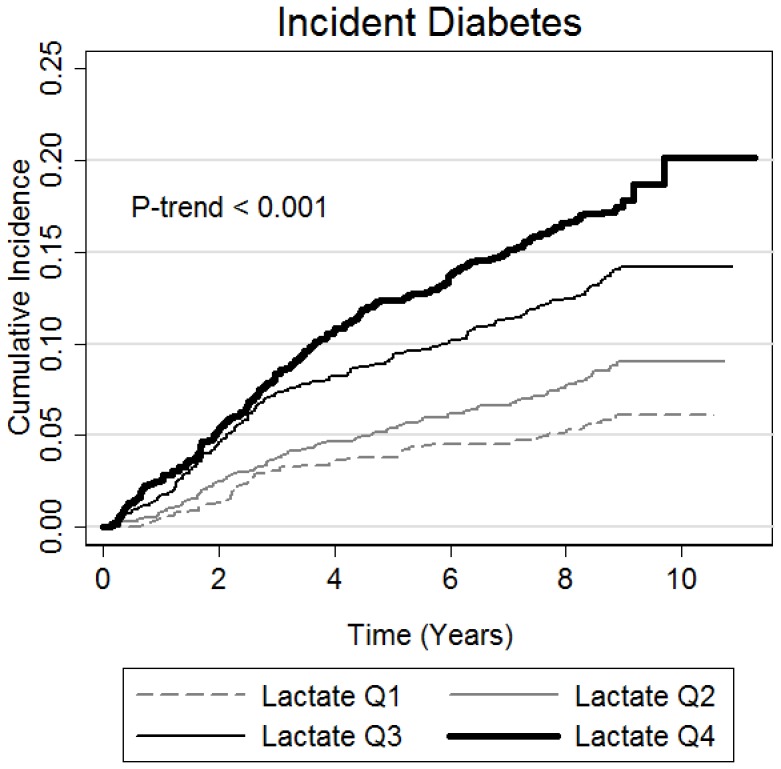
Kaplan-Meier cumulative incidence plot with follow-up years as the time axis and incident diabetes as the outcome stratified by baseline plasma lactate value. A Wald test was performed to assess for a trend across quartiles of lactate.

**Figure 3:**
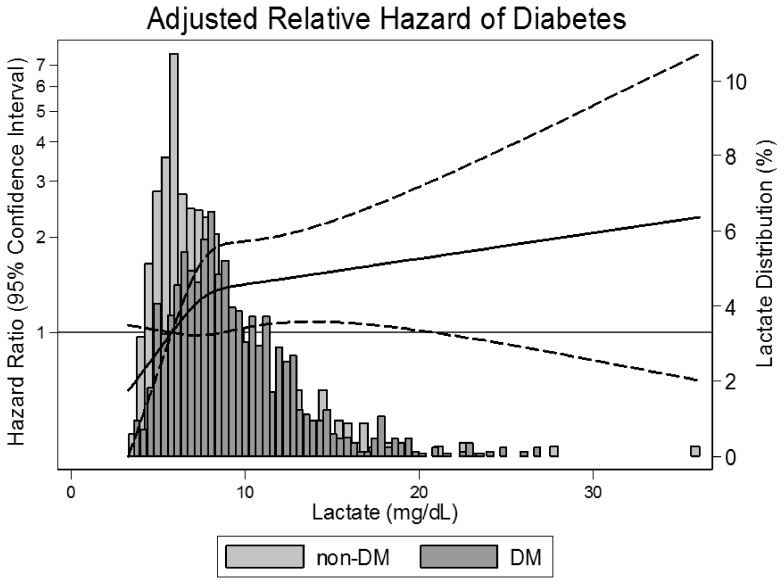
Histograms comparing the distribution of lactate in diabetic cases (DM) versus noncases (non-DM). The solid line represents a restricted cubic spline of the relative hazard with knots at 25^th^, 50^th^, and 75^th^ percentiles. The dashed lines represent the 95% confidence interval. Model is adjusted for age, sex, race, ARIC study center, education, hypertension status, history of coronary heart disease, smoking status, leisure index, parental history of diabetes, body mass index, waist circumference, triglycerides, low density lipoprotein cholesterol, and high density lipoprotein cholesterol.

There was a graded relationship between plasma lactate at baseline and the cumulative risk of incident type 2 diabetes over an average 7.9 years of follow-up (**Figure 2**). This graded relationship was robust after simultaneous adjustment for diabetes risk factors (**Table 3** **,** Models 1–4). In models adjusting for medical history, anthropometric variables and lipids, the association was attenuated but remained significant and strong (4^th^ quartile HR 2.05; 95% CI: 1.28, 3.28; *P*-trend ≤0.001). Histogram comparison revealed an upward shift in the distribution of lactate in diabetic cases versus non-diabetics (**Figure 3**). Similarly, a restricted cubic spline demonstrated an increase in the relative hazard for diabetes with increasing lactate levels, particularly below the 75^th^ percentile of the lactate distribution, i.e. a plasma lactate of 9.7 mg/dL.

**Table 3:** Hazard ratios (95% confidence intervals) for developing type 2 diabetes by weighted quartile of lactate concentrations.

	Quartile of Lactate				*P* for trend*
	≤5.8	5.8–7.3	7.3–9.7	>9.7	
Model 1	1.0 [Ref]	1.34 (0.89, 2.02)	2.13 (1.46, 3.10)	2.69 (1.84, 3.94)	<0.001
Model 2	1.0 [Ref]	1.19 (0.75, 1.89)	1.83 (1.21, 2.77)	2.41 (1.58, 3.68)	<0.001
Model 3	1.0 [Ref]	1.30 (0.79, 2.13)	1.45 (0.92, 2.29)	2.17 (1.38, 3.41)	<0.001
Model 4	1.0 [Ref]	1.27 (0.76, 2.13)	1.29 (0.80, 2.06)	2.05 (1.28, 3.28)	0.001
Model 5a	1.0 [Ref]	0.89 (0.51, 1.56)	0.78 (0.47, 1.31)	0.77 (0.44, 1.35)	0.45
Model 5b	1.0 [Ref]	1.05 (0.61, 1.81)	1.03 (0.64, 1.65)	1.46 (0.90, 2.39)	0.06
Model 5c	1.0 [Ref]	0.81 (0.46, 1.44)	0.70 (0.42, 1.18)	0.66 (0.37, 1.17)	0.26

Model 1: Age, gender, race, ARIC center, education.

Model 2: Model 1+ diagnosis of hypertension, prevalent coronary heart disease, smoking status, leisure index, parental history of diabetes.

Model 3: Model 2+ body mass index, waist circumference.

Model 4: Model 3+ log_10_ triglycerides, low density lipoprotein cholesterol, high density lipoprotein cholesterol.

Model 5a: Model 4+ fasting glucose.

Model 5b: Model 4+ log_10_ fasting insulin.

Model 5c: Model 4+ fasting glucose and log_10_ fasting insulin.

* P-value for trend evaluated using an ordinal variable based on the median lactate in each quartile.

Models examining the metabolic pathway between lactate and incident diabetes are shown in **Table 3**. The inclusion of fasting insulin as a covariate (Model 5b) attenuated the dose-response trend (4^th^ quartile HR 1.46, *P*-trend = 0.06), while the inclusion of fasting glucose (Models 5a & 5c), resulted in a non-significant reversal of the association (Model 5a: 4^th^ quartile HR 0.77, *P*-trend = 0.45; Model 5c: 4^th^ quartile HR 0.66, *P*-trend = 0.26).

A sensitivity analysis defining diabetes on the basis of diabetes medication use and self-report of physician diagnosis, i.e. excluding the subclinical cases identified by glucose measures, increased the magnitude of the association between lactate and incident diabetes (**Table S1**). In fact, even after adjusting for fasting insulin, lactate still demonstrated a significant trend across quartiles (*P*-trend = 0.02).

## Discussion

This analysis represents the largest population-based examination of the prospective relationship between lactate and type 2 diabetes. We observed a strong, graded relationship between plasma lactate and subsequent risk of incident type 2 diabetes over a 9-year follow-up period. The graded association, which was observed across the normal clinical range of lactate values, was independent of many traditional diabetes risk factors.

Prior to the current report there were several cross-sectional, clinical studies that found an association between lactate and insulin resistance [10], [11] as well as diabetes [9]. Furthermore, one prospective study of 766 Swedish men, found that an elevated lactate was associated with 2.4 times the risk of diabetes [13]. However, these studies were small and not readily generalizable. Strengths of our study include its large, biethnic community-based population of men and women with high quality measures of potential confounders.

In our study, we measured blood lactate levels in a population-based sample of resting subjects after an overnight fast and found that variations in the normal range (<18 mg/dl) were predictive of incident diabetes. Lactate is a product of glycolysis that rises markedly in states of hypoxia such as ischemia [22], with exercise [23], as well as with acute elevations in glucose and insulin [24], [25]. In contrast, plasma lactate in the rested, fasted state represents a balance between gluconeogenesis [26] and the rate of extrahepatic glycolysis [25]. A rise in resting lactate can be observed when gluconeogenesis is disrupted by alcohol use [27] or genetic disorders [28], [29]. Higher resting lactate concentrations may also be seen with increased extrahepatic glucose disposal or glycolysis [25] or with an increase in the concentration of lactate due to hypoxia [30], or oxidative insufficiency [31].

In the fasted, resting state, lactate is produced primarily by skeletal muscle and adipose tissue, and to a lesser extent by the brain, ethryrocytes, liver, gut, kidney, and skin [4], [25]. While skeletal muscle accounts for the majority of lactate release [24], a major determinant of variation in plasma lactate concentration is the rate of glucose disposal in extramuscular tissues [25]. This is exemplified by a comparison of obese and non-obese persons, in which greater lactate concentrations are due to increased lactate production from adipose tissue [24]. In addition to increased disposal, increased adiposity is associated with intracellular hypoxia [30] due to increased adipocyte size [32], [33] and inadequate vascularization [34]. Both these factors – excess glucose disposal and increased anaerobic glycolysis contribute to elevations in fasting lactate. However, we observed a relatively low correlation between BMI or waist circumference and lactate as well as a graded relationship between lactate and incident diabetes even after adjusting for measures of adiposity. This suggests that lactate is more than a marker of BMI or waist circumference.

There is strong evidence that oxidative insufficiency, evidenced by elevated plasma lactate concentrations, is associated with diabetes risk. Studies have found smaller mitochondrial size and lower density among adults with type 2 diabetes [35], [36] and individuals with insulin resistance [37]. Furthermore, there is evidence that mitochondria are less active in diabetic adults [36], [38]. Despite these associations, the temporal relationship between mitochondrial function and incident diabetes remains controversial: some argue that insulin resistance precedes and even induces mitochondrial inefficiency [39]. Since insulin signaling may improve oxidative capacity by increasing blood flow and by stimulating mitochondrial biogenesis, decreased oxidative capacity may be a consequence of decreased insulin signaling activity [40]. Decreased oxidative capacity is not universally observed in all patients with insulin resistance [41]. Furthermore, weight loss can decrease insulin resistance without impacting oxidative capacity and, among those at risk for type 2 diabetes, exercise can increase oxidative capacity without impacting insulin resistance [42]–[44].

In contrast to the work just described, there is strong evidence supporting a causal role for decreased oxidative capacity in the development of insulin resistance. Experimental manipulation of the mitochondrial genome in mice results in decreased oxidative capacity and insulin resistance [45]. Similarly, genetic mutations in human mitochondrial genes are associated with insulin resistance [5], [6] and diabetes mellitus [46], [47]. Several studies have also shown that decreased oxidative capacity precedes insulin resistance [48]. Moreover, exercise and weight loss [49] as well as insulin sensitizers like thiazolidinediones [50] improve insulin resistance, while exerting direct effects on oxidative capacity. Our study demonstrates that lactate as a marker of decreased oxidative capacity is associated with progression of insulin resistance. However, this was not independent of concurrent baseline measures of fasting glucose and insulin, suggesting that lactate does not represent a pathway distinct from insulin resistance. While establishing the temporal relationship between lactate and insulin resistance should be an important focus of future studies, our study suggests that studying the markers in concert rather than as individual causal factors may better reflect the underlying shift in metabolism that precedes insulin resistance and ultimately diabetes.

Another potential explanation for the relationship between lactate and incident diabetes is expression and activity of GLUT4, the glucose transporter in muscle and fat cells. In response to insulin, GLUT4 sequesters glucose into muscle and fat cells where it undergoes glycolysis [51], [52]. In certain settings, greater GLUT4 expression is associated with increased lactate production [53]. Furthermore, there is evidence that lactate itself can induce insulin resistance by suppressing GLUT4 expression [54]. Confirmation of this hypothesis is beyond the scope of this study, however.

Adjustment for insulin attenuated the association between lactate and incident diabetes, while adjustment for glucose non-significantly inverted the association. The change in direction of the association of lactate with incident diabetes after including glucose in models may be due to the role of fasting glucose in our case definition. When subclinical cases of diabetes were excluded from the outcome (**Table S1**), lactate was significantly associated with incident diabetes independently of insulin. Furthermore, lactate was no longer inversely associated with incident diabetes after adjustment for glucose, albeit non-significant. Whether this attenuation is due to loss in power from fewer cases or that lactate is not independent of glucose requires a larger study population.

Several limitations should be considered in the interpretation of these data. First, lactate was assessed with single plasma specimens that had been frozen for over 20 years. The effects of long-term storage on lactate concentration are unknown, and the lack of repeat measurements limit our ability to assess the within-person variability of lactate. Furthermore, this study is also limited with regard to the measurement of glucose and insulin. Although we attempted to explore glucose and insulin as mediators in the causal pathway of incident diabetes, our only lactate sample was measured at the same time as glucose and insulin. As a result, we were unable to establish temporality, which would be necessary to differentiate between their role as mediators or confounders of lactate and diabetes. Moreover, while we adjusted for many suspected confounders of the relationship between lactate and incident diabetes, it is possible we did not fully eliminate their effects. For example, while we adjusted for leisure index, a measure of physical activity, quantitative assessments of fitness and aerobic capacity were not performed. As a result, our models could be affected by residual confounding. Finally, it would have been informative to measure lactate during the visits between baseline and the development of diabetes as this could elucidate trends in lactate preceding and potentially contributing to incident diabetes.

In conclusion, our results show that lactate is a predictor of incident diabetes, suggesting a role for oxidative insufficiency in the pathogenesis of diabetes. Additional research is needed to determine the temporal relationship between elevated lactate and impaired fasting glucose and insulin resistance. Furthermore, future studies should attempt to elucidate the causal mechanism behind elevated lactate and diabetes pathogenesis.

## Supporting Information

Table S1 **Hazard ratios (95% confidence intervals) for developing type 2 diabetes by weighted quartile of lactate concentrations.** Type 2 diabetes defined by self-report or medication use alone (N = 233).
(DOCX)
