# Supplementary material for: Lactate and Risk of Incident Diabetes in a Case-Cohort of the Atherosclerosis Risk in Communities (ARIC) Study
Source: PLoS One. 2013 Jan 30;8(1):e55113. doi: 10.1371/journal.pone.0055113 (PMC3559502; doi:10.1371/journal.pone.0055113)
Supplement: Table S1 — Hazard ratios (95% confidence intervals) for developing type 2 diabetes by weighted quartile of lactate concentrations. Type 2 diabetes defined by self-report or medication use alone (N = 233). (DOCX) [file pone.0055113.s001.docx]

| **Appendix Table S1. Hazard ratios (95% confidence intervals) for developing type 2 diabetes by weighted quartile of lactate concentrations. Type 2 diabetes defined by self-report or medication use alone (N = 233).** | | | | | | |  |
| --- | --- | --- | --- | --- | --- | --- | --- |
|  |  | Quartile of Lactic Acid | | | |  |  |
|  |  | 1 (Low) | 2 | 3 | 4 (High) | *P* for trend* |  |
|  | Model 1 | 1.0 [Ref] | 1.49 (0.82, 2.70) | 2.31 (1.34, 4.00) | 3.47 (2.01, 5.97) | <0.001 |  |
|  | Model 2 | 1.0 [Ref] | 1.59 (0.81, 3.11) | 2.33 (1.27, 4.30) | 3.50 (1.90, 6.45) | <0.001 |  |
|  | Model 3 | 1.0 [Ref] | 1.73 (0.86, 3.50) | 1.85 (0.97, 3.52) | 3.13 (1.66, 5.90) | <0.001 |  |
|  | Model 4 | 1.0 [Ref] | 1.67 (0.81, 3.45) | 1.63 (0.85, 3.15) | 2.89 (1.51, 5.52) | 0.001 |  |
|  | Model 5a | 1.0 [Ref] | 1.29 (0.61, 2.73) | 1.12 (0.56, 2.26) | 1.29 (0.61, 2.74) | 0.64 |  |
|  | Model 5b | 1.0 [Ref] | 1.39 (0.65, 2.96) | 1.29 (0.66, 2.54) | 2.03 (1.03, 4.02) | 0.02 |  |
|  | Model 5c | 1.0 [Ref] | 1.15 (0.53, 2.47) | 0.98 (0.48, 1.99) | 1.07 (0.49, 2.30) | 0.97 |  |
| Model 1: Age, gender, race, ARIC center, education | | | | |  |  |  |
| Model 2: Model 1 + diagnosis of hypertension, prevalent coronary heart disease, smoking status, leisure index, parental history of diabetes | | | | | | | |
| Model 3: Model 2 + body mass index, waist circumference | | | | |  |  |  |
| Model 4: Model 3 + log10 triglycerides, low density lipoprotein cholesterol, high density lipoprotein cholesterol | | | | | | | |
| Model 5a: Model 4 + fasting glucose | | | |  |  |  |  |
| Model 5b: Model 4 + log10 fasting insulin | | | |  |  |  |  |
| Model 5c: Model 4 + fasting glucose and log10 fasting insulin | | | | |  |  |  |
| *P-value for trend evaluated using an ordinal variable based on the median lactate in each quartile. | | | | | | | |
